# Improving patient adherence to lifestyle advice (IMPALA): a cluster-randomised controlled trial on the implementation of a nurse-led intervention for cardiovascular risk management in primary care (protocol)

**DOI:** 10.1186/1472-6963-8-9

**Published:** 2008-01-14

**Authors:** Marije S Koelewijn-van Loon, Ben van Steenkiste, Gaby Ronda, Michel Wensing, Henri E Stoffers, Glyn Elwyn, Richard Grol, Trudy van der Weijden

**Affiliations:** 1 Maastricht University, School for Public Health and Primary Care, Department of General Practice, P.O. box 616, 6200 MD Maastricht, The Netherlands; 2 Radboud University Nijmegen, Centre for Quality of Care Research, Department of Quality of Care, P.O. Box 9101, KWAZO 114, 6500 HB Nijmegen, The Netherlands; 3 Department of Primary Care and Public Health, School of Medicine, Cardiff University, Neuadd Meirionnydd, Heath Park CF14 4YS, Cardiff, UK

## Abstract

**Background:**

Many patients at high risk of cardiovascular diseases are managed and monitored in general practice. Recommendations for cardiovascular risk management, including lifestyle change, are clearly described in the Dutch national guideline. Although lifestyle interventions, such as advice on diet, physical exercise, smoking and alcohol, have moderate, but potentially relevant effects in these patients, adherence to lifestyle advice in general practice is not optimal. The IMPALA study intends to improve adherence to lifestyle advice by involving patients in decision making on cardiovascular prevention by nurse-led clinics. The aim of this paper is to describe the design and methods of a study to evaluate an intervention aimed at involving patients in cardiovascular risk management.

**Methods:**

A cluster-randomised controlled trial in 20 general practices, 10 practices in the intervention arm and 10 in the control arm, starting on October 2005. A total of 720 patients without existing cardiovascular diseases but eligible for cardiovascular risk assessment will be recruited.

In both arms, the general practitioners and nurses will be trained to apply the national guideline for cardiovascular risk management. Nurses in the intervention arm will receive an extended training in risk assessment, risk communication, the use of a decision aid and adapted motivational interviewing. This communication technique will be used to support the shared decision-making process about risk reduction. The intervention comprises 2 consultations and 1 follow-up telephone call. The nurses in the control arm will give usual care after the risk estimation, according to the national guideline.

Primary outcome measures are self-reported adherence to lifestyle advice and drug treatment. Secondary outcome measures are the patients' perception of risk and their motivation to change their behaviour. The measurements will take place at baseline and after 12 and 52 weeks. Clinical endpoints will not be measured, but the absolute 10-year risk of cardiovascular events will be estimated for each patient from medical records at baseline and after 1 year.

**Discussion:**

The combined use of risk communication, a decision aid and motivational interviewing to enhance patient involvement in decision making is an innovative aspect of the intervention.

**Trial registration:**

Current Controlled Trials ISRCTN51556722

## Background

Despite a decreasing trend in mortality from cardiovascular diseases, these diseases are still the main cause of mortality in the Western world, and cardiovascular diseases cause 33% of all deaths in the Netherlands [1,2].

Unhealthy lifestyles, rather than medical conditions or genetic predisposition, are thought to be the most important and modifiable causes of the majority of deaths from CVD [3,4]. It is estimated that 30% of all new cases of coronary heart disease and 19% of all strokes are caused by smoking, while other lifestyle factors, such as diet, also play an important role in the development of CVD [5].

Preventive guidelines on CVD and diabetes recommend medical treatment for patients at high risk. In addition, they recommend patient education and counselling on smoking, diet, physical exercise and alcohol consumption for large groups of patients with moderately increased risk. The Dutch multi-disciplinary guidelines for cardiovascular risk management (CVRM) also underline the importance of a healthy lifestyle for the prevention of CVD. The core of the guideline consists of a risk table for the assessment of the 10-year risk of CVD, showing the various risk factors which can be reduced [6].

Lifestyle changing programmes for the prevention of CVD have shown moderate but significant effects and have proved to be cost-effective [7-12]. However, changing lifestyle is very difficult for people. The public's adherence to lifestyle advice and medication varies between 20 and 90%, with most estimates converging around 50% [13,14]. Improving this adherence requires effective interventions, comprising educational and behavioural components [15]. Adherence seems to be influenced by health beliefs such as risk perception, perceived benefits and disadvantages of treatment and self-efficacy, as well as stage of change and communication problems with physicians [16-18]. Each high-risk individual can usually choose between several options for risk reduction. Since patients seek treatment approaches which are manageable and in their view effective, their preferences can differ considerably from those of the professionals. Many people prefer lifestyle change to drug treatment, but beliefs about treatments differ between physicians and patients, making it difficult to achieve consensus about the best treatment. True dialogue is required if patients' preferences are to be used to make decisions effectively [19,20].

The IMPALA (IMproving Patient Adherence to Lifestyle Advice) project builds on earlier work at our department. Van der Weijden evaluated the effect of implementation of the Dutch cholesterol guideline for cholesterol among 6 GPs [21]. This resulted in negative findings, without improvement in underuse or overuse of statins. It appeared that the mismatch between the guideline and the patients' expectations or preferences was one of the main barriers to change. Van Steenkiste tried to involve patients in the decision-making process for cardiovascular risk reduction by implementing a decision support tool and using a risk table. He found no effect on the GPs' performance and a minimal effect in terms of the patients' cardiovascular risk reduction. From process evaluation it appeared that the patients had not been optimally exposed to the decision support tool. It was recommended delegating CVRM to a practice nurse and extending the implementation for involving the patient in CVRM [22]. For the IMPALA study, an innovative nurse-led implementation strategy was developed, comprising key elements of risk assessment, risk communication, a decision aid, and adapted motivational interviewing. The IMPALA study investigates whether patients' adherence to medication and lifestyle advice can be increased through patient involvement and shared decision making [20].

Many of the patients with moderate to high CVD risk, but without existing CVD, are monitored in primary care. According to the guideline for CVRM, prevention of CVD, especially patient education and support in lifestyle change, seems to be a good task to delegate to a practice nurse in primary care [6]. The number of general practices employing a qualified practice nurse is increasing. These practice nurses mainly perform standardised tasks for patients with chronic diseases like asthma, hypertension or diabetes. The nurse works under the supervision of a general practitioner.

Most patients have difficulty understanding CVD risks [22,23]. Perception of risks tends to be inaccurate and people find it difficult to interpret and act upon risk information. The format (framing) in which risk information is presented affects people's perception of risks and their decisions. For instance, information framed in terms of relative risk or loss framing is more persuasive than framing in terms of absolute risk or gain framing. This has been reported for both doctors and patients [24-29]. Risk communication should include weighing up the risks and benefits of a treatment choice, and should address the patient's perception of the probability of an event as well as the importance of the event for that individual [26].

Increasing people's competence to understand their actual cardiovascular risk, the pros and cons of possible treatments and their options for modifying the risk, as well as the competence to clarify their own values and to become involved in the decisions, seems to lead to better-informed decisions [18,30-32]. Information on treatment options and their advantages and disadvantages is ideally integrated in a well-designed and validated decision aid [33], which can help both physicians or practice nurses and patients in the process of making the best choice for the management of CVD risk. It may help the unaware high-risk person to agree with risk-reducing strategies and the 'worried-well' low-risk person to agree with non-indication for risk-reducing drugs. There is increasing evidence that decision aids increase patients' active involvement in decision making [32]. However, assessing the 10-year risk of CVD and informing the patient about different options for risk reduction is only one step in a process of lifestyle change. Additional components are needed to reach a shared decision which the patient is motivated to adhere to. Professionals will have to facilitate the process, and to communicate in a patient-oriented way.

The motivational interviewing method seems a good tool, in addition to the use of the risk chart and the decision aid, to achieve shared decision making [32]. Adapted motivational interviewing has been used [34] to set the agenda of consultations together with patients, to assess patients' motivations for behaviour change and build motivation for healthy behaviours, and to achieve goal setting and specific action plans. Motivational interviewing is an approach that can be described by its four basic principles: expressing empathy, developing discrepancy, rolling with resistance and supporting self-efficacy [34-36]. The consultations for the IMPALA intervention will be held in the spirit of these basic principles, and an adapted form of motivational interviewing will be used. Since it is possible that the two consultations will only be the first step in a long process of preventive support, a telephone consultation is added to the intervention as the initial step towards follow-up. Telephone counselling by practice nurses seems a valuable approach for adequate follow-up support [37,38].

The impact of the IMPALA intervention will be assessed by means of various evaluations. First, the effect of the nurse-led intervention on the adherence to lifestyle advice will be examined and compared with that of nurse-led usual care.

A more detailed understanding of the components of the intervention which are successful while others fail to change behaviour requires gaining insight into the 'black box' of the intervention. Prospective recording is a method to evaluate the intervention process in terms of materials used, themes of communication, and time needed. Studying interactions by means of audiotaped recordings can assess the use of motivational interviewing and the principles of shared decision making. This information will be used to examine factors that moderate or mediate intervention effects. Since attitudes play an important role in adapting to a new way of working [39], it is important to evaluate the experiences of the general practitioners and practice nurses with the intervention, to determine the feasibility of a wider implementation of the intervention.

The patients in the intervention arm will probably notice the attempts to promote patient involvement and the changes in the relationship between health professional and patient and in the communication during the consultations. In an earlier community-oriented and individual-oriented prevention project for cardiovascular diseases in the Dutch province of Limburg, frictions and irritations were found among both health advisors and patients in the consultations [40]. In this project, the research agenda had determined a large part of the agenda setting of the prevention activities, and the options for risk reduction were limited to certain themes. In the context of CVD prevention, a deliberative model of the relationship between physician or practice nurse and patient is preferable to other models of the physician-patient relationship [41]. In this model, the aim of physician-patient interaction is to help the patient determine and choose the best health-related values that can be achieved in the clinical situation. The role of the physician is that of a teacher or a friend, engaging the patient in a dialogue on the best course of action. The IMPALA project will involve interviews in which patients will be asked about their experiences with and opinions about the IMPALA intervention, so that this can be evaluated from an ethical perspective.

We hypothesise that the intervention will modify patients' adherence to lifestyle advice, but the intervention may also improve the healthcare process implemented by GPs and practice nurses and hence the amount of time and effort required from the professionals [42]. Since this study will determine both the effects and the costs of the implementation of the nurse-led intervention an economic evaluation of the intervention is planned to compare the cost-effectiveness ratio in the intervention group with that in the control group.

### Research questions

1. What is the effect of our nurse-led intervention for CVRM, including risk communication, a decision aid and adapted motivational interviewing, on patients' adherence to lifestyle advice? [Effect evaluation]

2. To what extent do the nurses adhere to the intervention for CVRM and decision support, and what are the experiences and opinions of GPs and practice nurses about this strategy? [Process evaluation]

3. How do patients experience the nurse-led intervention for CVRM, and what is the ethical value of the intervention? [Ethical evaluation]

4. What is the incremental cost-effectiveness ratio of the nurse-led intervention for CVRM and decision support compared to usual care by a practice nurse? [Economic evaluation]

## Methods

### Design

**Figure 1:**
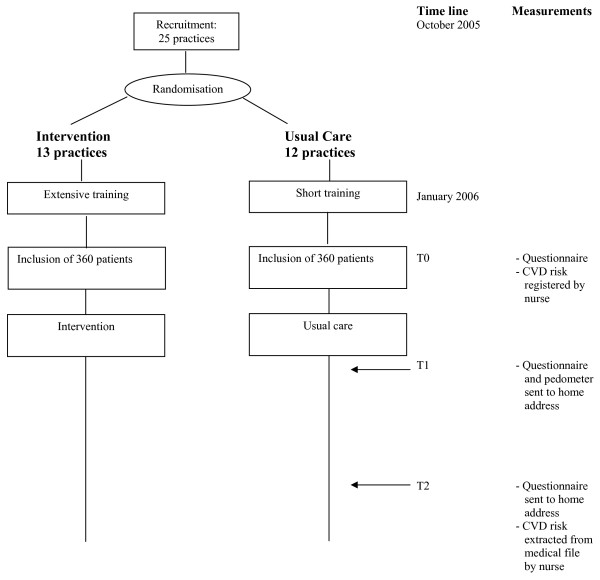
Flowchart of the IMPALA study.

The IMPALA study is a cluster-randomised controlled trial involving 20 general practices and 720 patients in the Netherlands. An independent statistician will perform a central block randomisation. Practices will be randomly allocated, 10 practices to the intervention group and 10 practices to the control group, after stratification into 4 geographical regions. Assessments will be made at the inclusion of the patients in the study, at 12 weeks and at 52 weeks. A flowchart of the study is shown in Figure 1.

### Ethical approval and informed consent

The Medical Ethical Committee of the University of Maastricht has granted ethical approval. The trial is registered as ISRCTN51556722 [43].

An information sheet will be given to all eligible patients. Patients will be informed on all aspects of the project by means of written information and personally at the inclusion. The privacy of the participating patients is protected, and all data will be coded and processed anonymously. It will be made clear in the informed consent form that each patient can terminate his or her participation the study at any moment. Patients will be asked to sign the informed consent form, and return this to Maastricht University to allow further contact regarding the research.

### General practices

Inclusion criteria for the general practices are that they have to employ a practice nurse and work with electronic patient records. Practices will be invited by letter to participate in the trial. Addresses will be obtained from the NIVEL (Netherlands Institute for Health Services Research) database. If a practice expresses interest, it will be visited by members of the project team for further information and explanation about the study. Non-responding GPs will be reminded after 1 month, by renewed postal invitation. The general practices will be located in the central and southern parts of the Netherlands. In the Netherlands, all patients are obliged to be registered with a general practice.

### Participants

The inclusion criteria for participants are based on the Dutch guideline for CVRM. According to the guideline, the following patients are eligible for a cardiovascular risk assessment:

Especially patients

• who have high blood pressure (≥ 140 mmHg) or are already being treated for it; and/or

• who have high total cholesterol (≥ 6.5 mmol/l) or already being treated for it; and/or

• who are smokers (men ≥ 50 years, women ≥ 55 years); and/or

• who have diabetes.

In addition, patients:

• who have positive family history of CVD; and/or

• who have visible obesity.

Patients with existing cardiovascular diseases, patients at high risk based on familial hypercholesterolaemia only and patients who are primarily managed in secondary care (e.g. by cardiologists or internists or in rehabilitation programmes) will be excluded from the study. This is a clinical approach to primary prevention. We will not manipulate the process of identifying potential high-risk patients other than by informing the GPs about the inclusion criteria, to stimulate usual strategies for case finding, either by the GPs during consultation hours or by the nurses inviting high-risk patients from available patient records. To facilitate the inclusion process, the GPs and practice nurses will receive a chart stand, with the inclusion criteria which they can place on their desk.

### Sample size calculation and feasibility of recruitment

The study will be powered on outcomes on patient behaviour to detect an absolute 52-week, post-intervention, between-group difference of 15% (50 – 65%) in patients' self reported adherence to life-style advice on smoking, diet, alcohol and physical exercise (alpha = 0.05, power = 0.80). The figure of 50% represents the average adherence by patients with chronic diseases [13]. The power calculation for clustered data analysis assumes an intra-class correlation coefficient (ICC) of 0.04. ICC values in earlier trials among general practitioners were 0.06 [44] and 0.03 [45]. Since we expect that the nurses will perform well in terms of adhering to the working protocol, we expect a lower ICC, with a maximum of 0.04. Thus, the within-cluster variance will be almost equal to the between-cluster variance. This implies that 600 patients are needed (300 per group) at 52 weeks. It is our intention that a total of 720 patients will be recruited by the GPs or practice nurses, to compensate for an expected 15% loss to follow-up (in an earlier trial by van Steenkiste, 87% of the included patients responded at the 6-month follow-up [45]). It should be possible to prospectively recruit 720 patients (36 per practice) in 6 months, as Dutch general practices each have 100–150 of these patients, who visit the practice at least once a year for regular monitoring.

### Intervention

**Figure 2:**
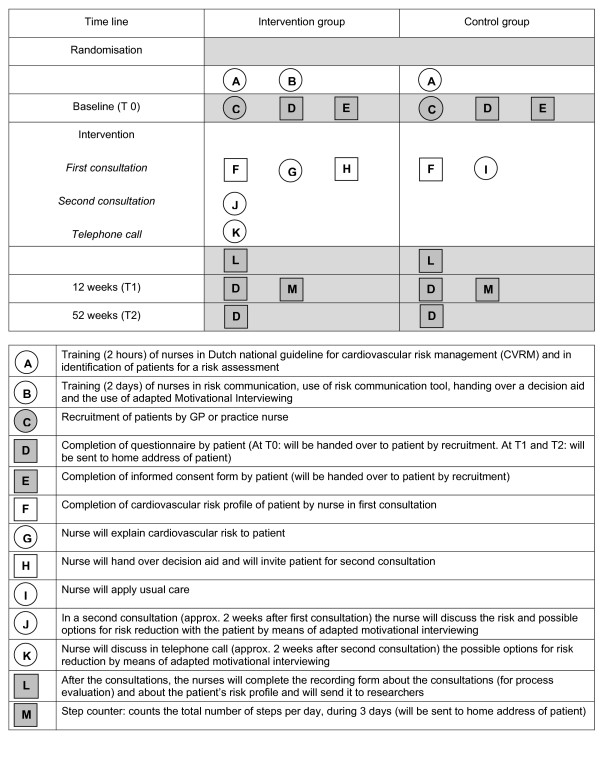
**Graphical depiction of intervention and measurements**. Graphical depiction of intervention and measurements (above), with legend (below). Squares represent fixed elements, e.g. objects and calculations. Circles represent activities that are flexible. Randomisation and measurements are shown in grey. This graphical method was proposed by Perera et al [76].

The GPs will delegate to the practice nurses the tasks of risk assessment, patient education and counselling. The practice nurses will be equipped to roll out an intervention comprising (1) risk assessment, (2) risk communication, (3) distribution of a decision aid and (4) motivational interviewing. These strategies will be distributed over two consultations and a follow-up telephone call (see Figure 2). Each consultation will take about 15 minutes, with a maximum of 30 minutes. The duration of the phone call will vary, but is not meant to last over 10 minutes.

#### (1) Risk assessment

The assessment of the 10-year risk of CVD will be done with the help of the risk table from the Dutch guideline on CVRM, published and produced in 2006 by the Dutch Institute for Health Care Improvement (CBO), in close collaboration with the Dutch College of General Practitioners (NHG), and has also been approved by the relevant clinical specialist groups, such as cardiologists [6,46]. The risk table is derived form the European Score risk table [46], adapted to the Dutch situation. The cardiovascular risk of patients with diabetes will be estimated with a table derived from the UKPDS risk engine [47].

#### (2) Graphical risk communication tool

The risk communication tool is a sheet showing graphical information that the nurse can use to communicate the risk more explicitly to the patient. It has been suggested that risks can be more understood by patients if different ways are used to present them, such as natural frequencies, positive and negative framing and a population chart. It has also been recommended to explain the uncertainty to the patient [48]. The practice nurse can visualise the total risk, as well as the relative contributions of modifiable risk factors to the total risk. Both absolute and relative risks will be framed and depicted in numbers (by natural frequencies) and in graphs (by population diagram). The risk communication tool was developed in a pilot study. A practice nurse from a general practice that will not be included in the main study administered the tool to 8 patients, who were interviewed about the different graphical risk presentations, clarity, language and layout of the tool. The tool was then adapted on the basis of the outcomes of these interviews.

#### (3) Decision aid

An earlier version of the decision aid was tested in an RCT by Van Steenkiste [45]. The 16-page decision aid (in brochure form) informs patients about their absolute 10-year CVD risk, both verbally and numerically, and presents options for risk reduction, such as use of medication or lifestyle changes. The risk table from the guideline on CVRM is also made available to the patients. The brochure will be handed over by the nurse to the patient at the end of the first consultation. The patient is expected to read the decision aid at home and return to the nurse to discuss what should or should not be done.

#### (4) Adapted Motivational Interviewing

In the second consultation, the nurse will apply adapted motivational interviewing to support the patient in the process of decision making about CVRM. The first step is to set the agenda for the consultation, together with the patient [36]. The patient will chose one or more key items in CVRM (smoking, physical exercise, dietary behaviour, alcohol consumption and adherence to medical treatment) in this agenda setting. After this, the nurse will assess the patient's current behaviour and motivation for change, by rating and exploring importance and confidence with respect to the chosen key items. If there is a need and sufficient motivation for change regarding one or more items, the nurse will consult with the patient to select one item as the goal for behaviour change. The nurse will inform the patient about local opportunities for individual or group support in lifestyle change that tie in with the patient's goal, if appropriate.

##### Follow-up

Little is known about the number of consultations featuring motivational interviewing that is needed to optimise the effect in terms of behaviour change, as regards intensity and frequency but the assumption is that a second motivational interviewing consultation will probably increase the effect of the intervention [49]. Therefore, the nurse will apply the same adapted motivational interviewing in the follow-up telephone call (lasting a maximum of 10 minutes). The nurse will assess the patient's actual motivations for change, ask for their experiences with specific actions (if applicable) and continue to work on importance and confidence.

The intervention can be embedded in a long-term treatment approach. After the telephone consultation, the nurse and the patient decide whether and what continuation is needed, for example some extra consultations to support the lifestyle change or regular monitoring of weight or blood pressure.

##### Training

The nurses will receive a two-day training, outside the practice. The GPs will only be invited for the part of the training that concerns the new guideline on CVRM. Homework about risk assessment and motivational interviewing will be sent to the nurses with the request to study the materials, which should take them about 4 hours. The two-day training consists of the following elements:

- Introduction 1: hour

- Outlining the multifaceted strategy and various aspects of the study: 1 hour

- Information on the new guidelines for CVRM, on the assessment of the individual 10-year risk of CVD by means of vignettes, on the recommendations for lifestyle counselling and risk reduction by medication such as blood pressure or cholesterol lowering drugs: 1.5 hours.

- Communicating the risk to patients using a risk communication tool, and disseminating the decision aid to patients: 2 hours.

- Specific training in the adapted motivational interviewing technique that is to be used to discuss the decision aid with patients after it has been studied by them at home; involving patients in decision making and goal setting: 7 hours.

Afterwards:

- After completing the training course, the nurses will audiotape 3 consultations and will receive written feedback from the researchers on their communication techniques.

### Usual care

The practice nurses for the control group will receive only a short training on the new guideline for CVRM and will be instructed to administer usual care consistent with current guidelines. Usual care depends on patients' risk profile and their preferences for risk reduction. In normal situations, when blood pressure and cholesterol levels are sufficiently controlled, the guidelines recommend one follow-up consultation a year. In order to keep the nurses in the control arm motivated, the extended training in risk communication and motivational interviewing will be offered after the inclusion period of the trial and after the short-term measurement at 12 weeks. To protect the contrast between the intervention group and the control group, the nurses for the control group have to 'promise' not to administer the intervention to the included patients before their last measurement (52 weeks). The nurses will receive a list of the included patients in their practice, specifying the exact moment of the last measurement for each patient.

#### Effect evaluation

The aim of the effect evaluation is to examine the patients' adherence to lifestyle advice. The research question is: *What is the effect of a nurse-led intervention for CVRM, including risk communication, a decision aid and adapted motivational interviewing, on patients' adherence to lifestyle advice?*

### Variables and measurements

Primary outcome measures are the patients' adherence to lifestyle advice and drug treatment. The current state-of-the-art in measurement of adherence behaviour is a multi-method approach that combines feasible self-reporting and reasonably objective measures [50,13]. Specific behaviours relating to smoking, saturated fat intake, fruit and vegetable consumption, physical exercise, alcohol use and use of cardiovascular medication will be reported by patients, using validated self-reported questionnaires. For a detailed overview of the outcome measures, questionnaires and types of variables, see Tables 1 and 2. We will use pedometers at 12 weeks to measure physical exercise [51], although we recognise that this measurement may itself increase activity levels. Pedometers are reasonably accurate for walking activities [50]. Patients will be instructed to measure during 3 days in one week [52]. Body mass index will be measured as a proxy measure of healthy diet and exercise.

**Table 1:** Primary outcome measures

**Outcome measure**	**Instrument **(Number of items, reference, validated yes/no)	**Analysis**
Fruit and Vegetables	8 items; [66]; Validated questionnaire	mean sum score; proportion meeting recommendations
Fat intake	35 items; [67] Validated questionnaire	mean sum score
Physical exercise	15 items; [68, 69]; validated questionnaire, modified Dutch version of the CHAMPS has not been validated.	mean; proportion meeting recommendations
Smoking	2 items; [70]; Validated questionnaire	proportion smoking
Alcohol consumption	2 items; [71]; validated questionnaire	proportion meeting recommendations
Adherence to medical treatment	5 items; (MARS) [72], not validated	mean
Cholesterol level	[6] Measured by nurse	mean score, proportion above recommendation
Blood pressure	[6] Measured by nurse	mean score, proportion above recommendation
Body Mass Index	[6] Measured by nurse	mean score, proportion above recommendation
10-year risk of CVD	based on patient's sex, age, blood pressure, cholesterol and smoking behaviour; [73];	mean score, proportion above recommendation

**Table 2:** Secondary outcome measures

**Outcome measure**	**Instrument **(Number of items, reference, validated yes/no)	**Analysis**
Perception of own health behavior	1 item for each primary lifestyle outcome; question with 5-point scale	mean
Attitude towards behaviour change	1 item for each primary lifestyle outcome; question with 5-point scale	mean
Self-efficacy about specific behaviour change	1 item for each primary lifestyle; question with 5-point scale	mean
Risk perception	2 items; [74]	mean
Anxiety	2 items; [45]	proportion
Satisfaction with communication and confidence in decision	20 items; (COMRADE) [75]	mean

Clinical endpoints will not be assessed, but the absolute risk of cardiovascular events in 10 years will be calculated using the risk table from the Dutch guideline for CVRM [6]. For patients with diabetes, the UK PDS risk engine will be used. Calculation of the patients' absolute risk of cardiovascular events in 10 years, which is a proxy measure of actual health gain, will be based on known and available determinants: diabetes, age, sex, smoking, blood cholesterol level and systolic blood pressure. After one year, the risk of CVD will also be estimated, by deriving data on the risk factors from medical records in general practice. Since there is (according to the CVRM guidelines) no need to assess someone's cholesterol level annually if this level is acceptable, it is possible that some of the data used for the estimation will not be recent (with a maximum of one year).

Secondary outcome measures are the perception of the patients' own health behaviour, risk perception, anxiety, self-efficacy, importance of behaviour change, satisfaction with communication and confidence in the decision that has been made. Other factors that will be recorded include patient demographic factors (age, sex, education) and medical characteristics. See Table 2 for secondary outcomes.

### Timing of measurements

Measurements will take place at three moments (see Figures 1 and 2):

- T0 (baseline): The patients will receive the first questionnaire from their GP or practice nurse at the time they are invited to enrol in the study. The nurse will send a postage-paid postcard with the patient number to the researchers at Maastricht University (UM). Before a patient visits the practice for the first consultation, he/she will complete the first questionnaire and an informed consent form. After each consultation, the nurse will record on a standardised form some patient characteristics like age, sex, blood pressure, cholesterol level, glucose level, height, weight and the occurrence of CVD in the family (yes or no).

- T1 (12 weeks): A second questionnaire and the pedometer will be sent to the patient's home address.

- T2 (52 weeks): The final questionnaire will be sent to the patient's home address. The nurses will be asked to record data regarding the biological parameters (cholesterol level, blood pressure, glucose, diabetes yes/no) from the most recent patient contact.

All the questionnaires, the informed consent form and a form to record the number of steps that were made are to be sent to the researchers in a postage-paid envelope after completion. Materials (questionnaires, informed consent form, recording forms for nurses, initial postcard) will carry a unique patient number.

### Data analysis

Since it is not possible to restrict our study to one primary outcome for all patients beforehand, the study will examine 6 different lifestyle changes: smoking, physical exercise, fat intake, fruit, vegetables and alcohol.

To assess the difference in primary outcomes between the arms, the arms will first be compared in terms of changes in the specific lifestyle issues, independent of the targets chosen by the intervention patients. We will use a T test for continuing variables and a χ^2 ^test for dichotomous variables.

A second analysis of primary outcomes is a multilevel regression analysis (using SPSS and MLwin), which uses adherence to the primary outcomes of lifestyle change as the dependent variables and group allocation and pre-intervention scores as independent variables. Additional independent variables will be included in the model, such as patient characteristics (age, sex, anxiety and risk of CVD). Additional regression analyses will focus on the effect at T2.

The third analysis involves constructing a simple standardised overall adherence score for the six lifestyle items, which expresses to what extent, on average, patients achieved the behaviour change goals of the national recommendations for a healthy lifestyle. The score of the intervention group will be compared with that of the control group.

Finally, the reduction in the 10-year risk of CVD will be analysed for differences between the two arms. The risk of CVD will be analysed with a multilevel regression analysis, which uses the risk at 52 weeks as the dependent variable and group allocation and pre-intervention risk as independent variables.

The secondary outcomes will also be analysed for the differences between the arms, using a T test for continuing variables and a χ^2 ^test for dichotomous variables. We also intend to use a multilevel regression analysis (using MLwin) which uses e.g. self-efficacy and attitudes at T1 as the dependent variables and group allocation and pre-intervention scores as independent variables. Additional independent variables, such as patient characteristics (age, gender, risk of CVD and socio-economic status), will also be included in the model. Additional regression analyses will focus on the effect at T2. Further analyses for subgroups of patients are also possible.

In addition, we will describe the personal targets of the patients in the intervention arm, which will be recorded by the nurses.

#### Process evaluation

The purpose of the process evaluation is to establish actual exposure to the intervention as it was intended, and to examine which components of the intervention were successful and which ones were not. The research question is: *To what extent do the nurses adhere to the intervention for CVRM and decision support, and what are the experiences and opinions of the GPs and nurses with regard to this intervention?*

### Variables and measurements

Data on the nurses' actual adherence to the components of the strategy will be gathered by the nurses themselves by means of prospective self-reports. Nurses will use a short standardised questionnaire after each consultation to record which components of the intervention have been applied. Each item will be scored as a done/not done binary variable (see Table 3 for the key intervention features that will be scored). On top of this, each nurse will be instructed to audiotape 3 consultations, namely one of the initial ones and two later on in the trial. In addition to this quantitative approach to process evaluation, we will also use a qualitative approach, in that the nurses will be invited to a focus group interview to discuss their perception of determinants of success and failure. The variation in the effects at the patient and nurse levels will also be discussed. Finally, a subgroup of GPs will be invited for face-to-face interviews about their opinion on the intervention. The sample will include practices with successful as well as less successful patient inclusion. The focus group and the interviews will be moderated by an experienced GP, who is not a member of the research group.

**Table 3:** Key features of the intervention for quantitative process evaluation

**1^**st **^consultation**	Nurse explains the risk to the patient by means of the risk communication tool.
	Nurse hands over the risk communication tool.
	Nurse hands over decision aid booklet + risk communication tool (to consider at home).
**2^**nd **^consultation**	Patient shows up for second consultation. If patients cancel, they are asked for the reason
	Nurse uses motivational interviewing; sets agenda with the help of an agenda-setting chart, establishes importance and confidence, explores importance and builds confidence by asking the patient questions.
	Which options for risk reduction were discussed during the consultation?
	Nurse guides the patient in formulating the main personal goal for lifestyle change (if applicable).
	When medication is prescribed: has the nurse consulted the GP?
	Which other health education materials were used during the consultation?
**Telephone call**	The telephone call takes place. If patients cancel, they are asked for the reason
	Nurse uses motivational interviewing; sets agenda with the help of an agenda-setting chart; establishes importance and confidence, explores importance and builds confidence by asking the patient questions.
	Which options for risk reduction were discussed during the telephone call?
	Nurse guides the patient in formulating the main personal goal for lifestyle change (if applicable).
**Extra items**	Time needed per patient contact.
	Time needed to discuss patients with GP.
	Appointment for follow-up consultation after the telephone call, if necessary.

### Data analyses

The self-reported done/not done components of the intervention will be presented as the proportion of the consultations in which the component has been applied. The audiotaped consultations in the intervention group will be scored with the IMPALA Practice nurses Scoring list (IPS), a scoring list based on the IMPALA training programme and on other existing scoring lists [53,54], to determine the extent to which the motivational interviewing technique was implemented. The consultations will be scored with the OPTION instrument to measure to what extent practice nurses have involved patients in the decision-making process [55,56]. The interviews with the nurses and the GPs will be transcribed and coded by three persons, using a coding list. Differences of opinion will be discussed until agreement is reached.

#### Ethical evaluation

The ethical evaluation will use interviews with individual patients regarding their opinions about and experiences with the consultations of the intervention, to evaluate the intervention in the light of the models of physician-patient relationship by Emanuel and Emanuel [41].

The research question is: *How do patients experience the nurse-led intervention for CVRM, and what is the ethical value of the intervention?*

### Variables and measurements

At least 16 patients of 4 practices will be invited by the nurses to participate in an interview. The 4 nurses implementing the intervention most satisfactorily (judging from the audiotaped consultations), will be asked to invite a variety of patients in terms of sex, 10-year risk of CVD and the course of the consultations.

The interviews will be 'open', in that the interviewer will try to access the patient's personal story. The interviews will be held in the diagnostic style, and will characterised by an interested and personal approach [57]. The interviews will be based on a list of themes to ensure that all relevant items are brought up during the discussion (see Table 4). All interviews will be audiotaped, with the patients' permission, and transcribed later. The interviews will be done by an experienced junior social scientist, who is not a member of the research group.

**Table 4:** Interview themes for ethical evaluation

1. Expectations before the consultations about:	the role of the patient (e.g. active/passive);
	the role of the practice nurse (e.g. guiding, informative, clarifying, reflecting);
	the gain from the consultations (e.g. information, becoming aware, getting attention, reflection and a motivated decision, guiding towards a decision).
2. Experiences with the consultations	Understanding the situation.
	What had the patient picked up from the consultations?
	What was the role of the tools in this understanding?
	How was the patient involved in the consultations?
	Active participation in the discussion.
	Personal involvement in the topic.
	Contribution to the decisions that were made.
	How did the nurse approach the patient (e.g. attentive, open to the patient's opinion, steering, informative, giving his/her own view)?
	What was the impact of the consultation
	for the patient's personal life and well-being;
	as regards clear decisions, goals and a plan?
	The tools of the intervention
	What did the tools mean to the patient?
	Was anything missing from the consultations?
3. Preferences for future consultations:	about the patient's role;
	about role of the practice nurse;
	about the significance of the consultation. When is a consultation successful?

### Data analysis

The interviews will be analysed by the constant comparative method as described in grounded theory [58]. Interviews will be coded and the codes will be grouped, compared and categorised.

#### Economic evaluation

The aim of the economic evaluation is to assess the costs of the IMPALA intervention. The research question is: *What is the incremental cost-effectiveness ratio of a nurse-led intervention for CVRM and decision support compared to usual care by a practice nurse?*

### Measurements and variables

The use of CVD-related health care resources will be measured by means of questions about health care use (prescribed medication, tests, referrals, numbers of contacts) over a 3-month period before completion. Costs of CVD events will be estimated from the literature and/or analyses of aggregate data sources (hospital information systems). Cost calculation will be based on real prices or on unit prices from the Dutch guideline for cost calculation [59]. Quality of life will be measured with the SF-36, a validated questionnaire [60]. The costs of implementing the intervention will be recorded by the researchers.

### Timing of measurements

The questions for the economic evaluation will be incorporated in the questionnaires of the effect evaluation at T0, T1 and T2.

### Data analyses

The economic evaluation will be a cost-effectiveness study. We expect no differences in short-term societal costs because the improvements in lifestyle will mainly have effects in the long term. Therefore, the analyses will use a health care sector perspective, and will thus also show the expected costs and consequences per general practice. The base case involves calculation of an incremental cost-effectiveness ratio (ICER) of patient involvement as regards the estimated reduction of CVD events in 10 years on the basis of T2 data [61]. Quality improvement cost-effectiveness will be combined with treatment cost-effectiveness according to the Mason model [62,63]. Cost differences between the intervention and control groups will be statistically tested by calculating the 2.5 – 97.5 percentile intervals of the bootstrapped resamples.

In addition, we will use a long-term economic evaluation to explicitly reveal the relationship between the CVD risk reduction achieved and the required investment for the implementation strategy. For this purpose, a Markov health state transition model will be developed, with a first-order Monte Carlo simulation in which each patient goes through the model individually. The cycle length will be one year. The time horizon of the analysis is the full lifetime of the population. Costs and benefits will be discounted according to national guidelines (4%). A one-way sensitivity analysis will be used to establish the individual effects of model parameters on the results of the analysis. The model parameters will be varied across a plausible range. Parameter uncertainty will be further tested using probabilistic sensitivity analysis, which takes into account the fact that some combinations of factors are more likely than others [64].

## Discussion

The intervention that will be evaluated in this trial is characterised by some innovative aspects. Firstly, not much is known yet on the effect of the combined use of risk communication, a decision aid and motivational interviewing to enhance patient involvement in decision making. Secondly, delegation of such tasks to a practice nurse is a new approach in the Netherlands, and is in line with current changes in general practice care processes in this country (increasing practice sizes and multidisciplinary health centres employing nurses).

A factorial or multiple-arm RCT design would have been optimal from the methodological perspective, and could shed light on the effectiveness of the individual ingredients of this multifaceted intervention in terms of the primary outcomes. However, this would have blown up the design to include multiple trial arms and large numbers of practices and patients, and turn it into an unfeasible experiment. The analyses of our secondary outcomes will probably shed light on the effectiveness of individual ingredients of the intervention. In some of the secondary outcomes, such as risk perception, the relation with individual ingredients, such as risk communication, seems one-dimensional. The way in which risk communication, the decision aid and motivational interviewing are applied during the trial will be documented in a process evaluation, which will give further information on the impact of intervention components on the main outcomes.

The pilot version of the risk communication tool included a Paling scale, a scale to compare the risk of cardiovascular risk with other risks of life. After the pilot test, we decided to remove the Paling scale because it was too difficult according to the patients who received the risk communication tool in this pilot test.

When we developed the decision aid, the IPDAS criteria for decision aids had not yet been published [65], but the decision aid does comply with most of the these criteria, namely using a systematic development process, providing information about options, presenting probabilities, disclosing conflicts of interests and using plain language. On the other hand, our decision aid does not fully meet the criteria on balancing the presentation of options, clarifying and expressing values, and guiding/coaching in deliberation and communication, although patients will be invited to deliberate about the decision making, and the motivational interviewing in our strategy is meant to help patients clarify and express values.

Cardiovascular risk is not the primary outcome of the effect evaluation because this is a pragmatic trial about the implementation of the Dutch national guideline for CVRM. To reduce the burden of data collection for the health care professionals, we will ask them to collect only the data they need for the risk estimations. There is no budget for additional data collection outside routine practice by the researchers.

Although we intended to use more objective measures for the primary outcomes of the effect evaluation, this was precluded by logistic and financial reasons. The relatively inexpensive breath carbon monoxide test is not very sensitive, and the more reliable urine cotinine marker is expensive and difficult from a logistic point of view. A biomarker for fruit and vegetables, such as carotene measurement, is expensive, and no simple biomarker for alcohol is available. The influence of information bias resulting from subjective self-reports is reduced in the data analysis, which takes the pre-intervention scores (which have the same information bias) into account. Although self-reported smoking status is known to be biased, measurement of a biological marker has various disadvantages.

## Abbreviations

CVRM: cardiovascular risk management

CVD: cardiovascular diseases

GP: general practitioner

NIVEL: the Netherlands Institute for Health Services Research. Nivel contributes to the body of scientific knowledge about the provision and use of health care services. One of Nivel's responsibilities is to register all general practitioners in the Netherlands.

UK PDS risk engine: a digital calculation tool for the estimation of CVD in patients with diabetes mellitus, based on the UK Prospective Diabetes Study.

## Competing interests

The author(s) declare that they have no competing interests.

## Authors' contributions

MK is a health scientist, main investigator and PhD student. She is involved in developing the intervention and the instruments, as well as in the implementation, analysis and reporting aspects of the trial. TvdW is an epidemiologist. She is the project leader and is involved in all aspects of the study. BvS is a health scientist and is involved in all aspects of the study, especially as regards the decision aid, instruments, analyses and economic evaluation. GR is a health scientist and senior researcher, involved in all aspects of the effect and process evaluation, especially motivational interviewing and instruments to measure lifestyle. RG is PhD supervisor and psychologist and involved in the design of the study, the analyses and reporting. GE is PhD supervisor; he is a GP and advises the project team on shared decision making. MW is a medical sociologist and advises the project team on intervention, measurement instruments, design and economic evaluation. HS is a GP and is involved in the design of the study. All authors have read and approved the final version of the manuscript.

## Pre-publication history

The pre-publication history for this paper can be accessed here:


